# Venetoclax–based low intensity therapy in molecular failure of *NPM1*-mutated AML

**DOI:** 10.1182/bloodadvances.2023011106

**Published:** 2023-12-02

**Authors:** Carlos Jimenez-Chillon, Jad Othman, David Taussig, Carlos Jimenez-Vicente, Alexandra Martinez-Roca, Ing Soo Tiong, Manish Jain, James Aries, Seda Cakmak, Steven Knapper, Daniel Tuyet Kristensen, Vidhya Murthy, Joy Zacharoula Galani, Charlotte Kallmeyer, Loretta Ngu, David Veale, Simon Bolam, Nina Orfali, Anne Parker, Cara Manson, Jane Parker, Thomas Erblich, Deborah Richardson, Katya Mokretar, Nicola Potter, Ulrik Malthe Overgaard, Anne Stidsholt Roug, Andrew H. Wei, Jordi Esteve, Martin Jädersten, Nigel Russell, Richard Dillon

**Affiliations:** 1 Servicio de Hematología y Hemoterapia, Hospital Universitario Ramón y Cajal, Madrid, Spain; 2 Department of Medical & Molecular Genetics, King’s College London, London, United Kingdom; 3 Guy’s and St Thomas Hospital, London, United Kingdom; 4 Faculty of Medicine and Health, The University of Sydney, Sydney, Australia; 5 Department of Haematology, Royal Marsden Hospital, Sutton, United Kingdom; 6 Institut d’Investigacions Biomèdiques August Pi i Sunyer, Barcelona, Spain; 7 Hematology Department, Hospital Clínic Barcelona, Barcelona, Spain; 8 Peter MacCallum Cancer Centre, Royal Melbourne Hospital and Walter and Eliza Hall Institute of Medical Research, Melbourne, VIC, Australia; 9 Alfred Hospital and Monash University, Melbourne, VIC, Australia; 10 Austin Health and Olivia Newton John Cancer Research Institute, Melbourne, VIC, Australia; 11 Department of Haematology, Leeds Teaching Hospitals Trust, Leeds, United Kingdom; 12 Department of Haemato-Oncology, St Bartholomew’s Hospital, London, United Kingdom; 13 Department of Haematology, School of Medicine, Cardiff University, Cardiff, United Kingdom; 14 Department of Haematology, Clinical Cancer Research Center, Aalborg University Hospital, Aalborg, Denmark; 15 Department of Haematology, University Hospitals of Birmingham, Birmingham, United Kingdom; 16 Department of Haematology, Dartford & Gravesham NHS Trust, Dartford, United Kingdom; 17 Department of Haematology, Lincoln County Hospital, Lincoln, United Kingdom; 18 Department of Haematology, Royal Devon University Healthcare NHS Foundation Trust, Exeter, United Kingdom; 19 Department of Haematology, Taunton and Somerset NHS Foundation Trust, Taunton, United Kingdom; 20 Department of Haematology, St. James's Hospital, Dublin, Ireland; 21 Department of Haematology, Queen Elizabeth University Hospital, Glasgow, United Kingdom; 22 Department of Haematology, Northampton General Hospital, Northampton, United Kingdom; 23 Department of Haematology, The London Clinic, London, United Kingdom; 24 Department of Haematology, University Hospital Southampton, Southampton, United Kingdom; 25 Synnovis, London, United Kingdom; 26 Department of Haematology, Rigshospitalet, Copenhagen, Denmark; 27 Department of Haematology, National Hospital, Copenhagen, Denmark; 28 Department of Hematology, Aarhus University Hospital, Aarhus, Denmark; 29 Department of Medicine, Center for Haematology and Regenerative Medicine, Karolinska Institutet, Stockholm, Sweden; 30 Department of Haematology, Karolinska University Hospital, Stockholm, Sweden

## Abstract

Molecular failure in *NPM1*-mutated acute myeloid leukemia (AML) inevitably progresses to frank relapse if untreated. Recently published small case series show that venetoclax combined with low-dose cytarabine or azacitidine can reduce or eliminate measurable residual disease (MRD). Here, we report on an international multicenter cohort of 79 patients treated for molecular failure with venetoclax combinations and report an overall molecular response (≥1-log reduction in MRD) in 66 patients (84%) and MRD negativity in 56 (71%). Eighteen of 79 patients (23%) required hospitalization, and no deaths were reported during treatment. Forty-one patients were bridged to allogeneic transplant with no further therapy, and 25 of 41 were MRD negative assessed by reverse transcription quantitative polymerase chain reaction before transplant. Overall survival (OS) for the whole cohort at 2 years was 67%, event-free survival (EFS) was 45%, and in responding patients, there was no difference in survival in those who received a transplant using time-dependent analysis. Presence of *FLT3*-ITD mutation was associated with a lower response rate (64 vs 91%; *P* < .01), worse OS (hazard ratio [HR], 2.50; 95% confidence interval [CI], 1.06-5.86; *P* = .036), and EFS (HR, 1.87; 95% CI, 1.06-3.28; *P* = .03). Eighteen of 35 patients who did not undergo transplant became MRD negative and stopped treatment after a median of 10 months, with 2-year molecular relapse free survival of 62% from the end of treatment. Venetoclax–based low intensive chemotherapy is a potentially effective treatment for molecular relapse in *NPM*1-mutated AML, either as a bridge to transplant or as definitive therapy.

## Key Points

• 56 out of 79 (84%) patients treated with VEN-combinations for *NPM1* molecular failure achieved an MRD response, and 71% became MRD negative.
• Venetoclax combinations are a potentially effective treatment for molecular failure, either as a bridge to transplant or as definitive therapy.

## Visual Abstract

**Figure:**
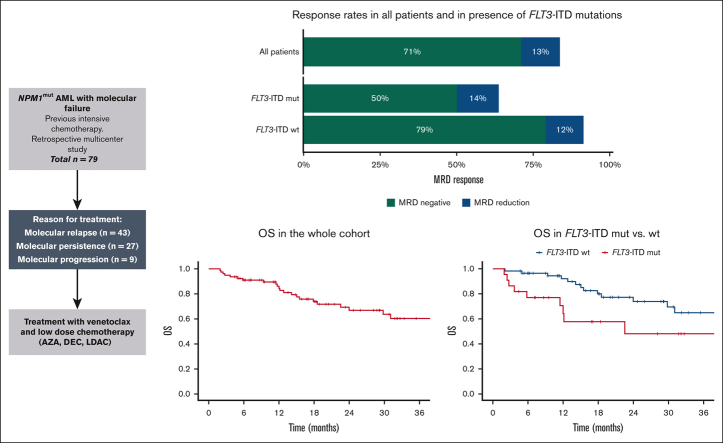

## Introduction

Nucleophosmin (*NPM1*) mutations (*NPM1*^mut^) are present in approximately one-third of adults with acute myeloid leukemia (AML),^1^ and despite a generally favorable prognosis, a significant proportion (26%-44%) will relapse.^2^, ^3^, ^4^ Importantly, *NPM1*^mut^ provides a stable target for monitoring measurable residual disease (MRD) using molecular methods,^5^ and patients with rising MRD levels after treatment (now called MRD relapse^6^) inevitably progress to frank relapse without intervention.^7^^,^^8^ Although transplantation may play an important role for eligible patients, proceeding to transplant with high levels of MRD appears to be associated with poor outcomes.^9^, ^10^, ^11^

There are currently limited data regarding interventions for molecular failure, and treatment options are not well defined. In the RELAZA2 trial, 17 of 32 patients (55%) with *NPM1*^mut^ AML treated with azacitidine at MRD relapse achieved MRD negativity.^12^ More recently, 2 retrospective studies using venetoclax and azacitidine or low-dose cytarabine (LDAC) in this situation reported MRD negativity in 11 of 12 (92%) and 9 of 11 patients (82%),^13^^,^^14^ and a number of patients in both studies subsequently received an allogeneic transplant. Because of the convenience and low toxicity of these regimens compared with salvage chemotherapy (SC) and the lack of alternatives, off-label use of venetoclax combinations in this situation has become common in several European countries. Here, we present outcomes in a large international real-world cohort of patients with *NPM1*^mut^ AML and MRD relapse or persistence treated with venetoclax combinations.

## Methods

### Patients

Patients with AML with an *NPM1* mutation (of any type) who had received venetoclax combinations for molecular failure were retrospectively identified from 20 hospitals in the United Kingdom, Sweden, Australia, Spain, Denmark, and Ireland between May 2017 and October 2022. The inclusion criteria were as follows. Patients had to be aged ≥16 years with a diagnosis of AML according to World Health Organization 2016^15^ with an *NPM1* mutation at diagnosis. They had to have received anthracycline-based induction chemotherapy as firstline (supplemental Figure 1) and had molecular failure diagnosed in 1 of 5 central reference laboratories, which was defined as follows. Patients either had MRD relapse as defined by European LeukaemiaNet 2022 (ie, either conversion from MRD negativity to positivity confirmed on a second sample, molecular relapse; or a confirmed 1-log_10_ rise in transcript expression, molecular progression)^6^^,^^16^ or had persistent MRD at the end of treatment (EOT; ie, molecular persistence) and at least 1 risk factor for progression (*FLT3*-ITD or EOT *NPM1*^mut^ MRD < 4.4-log reduction).^17^ Patients had to have at least 1 posttreatment bone marrow sample evaluable for MRD response assessment by reverse transcription quantitative polymerase chain reaction. Patients showing hematologic or extramedullary relapse before treatment and those treated with high intensity venetoclax–based regimens were excluded from this study. Twelve patients from a previous publication were also included in this cohort.^13^ *FLT3* mutational status was assessed at diagnosis in accredited diagnostic laboratories. This study was approved by local ethics committees in accordance with the Declaration of Helsinki. Informed consent was waived for this retrospective study.

### Treatment

Patients were treated under institutional protocols using off-label venetoclax (100-600 mg taken orally daily for 7-28 days) in combination with azacitidine (75-100 mg/m^2^ subcutaneous [SC] daily for 5-7 days), LDAC (20 mg/m^2^ SC daily for 7-10 days), or decitabine (20 mg/m^2^ IV daily for 5 days). Patients proceeded to allogeneic stem cell transplantation or ceased treatment at the discretion of the treating physician.

### MRD assessment

Patients were routinely monitored by reverse transcription quantitative polymerase chain reaction for mutant *NPM1* transcripts using bone marrow aspirate samples (except for 1 patient monitored by DNA assay due to a rare *NPM1* mutation). Complementary DNA was prepared from total RNA, and *NPM1*-mutated transcripts were amplified with mutation-specific primers as previously described.^8^^,^^18^ *NPM1* mutant transcript levels were compared with the expression of the *ABL1* reference gene. Quantitation was performed with reference to a standard curve of serially diluted plasmid standards (Qiagen). Assay sensitivity varied between patients and samples but was generally in the range of 1:10^–5^ to 1:10^–6^. No data on multiparametric flow cytometry were obtained for this study.

### Response definitions

The following response definitions were used. MRD negativity required amplification of *NPM1* mutated transcripts in fewer than 2 replicates out of 3, using a cycle threshold (Ct) cutoff of 40, in a sample with adequate sensitivity indicated by a median *ABL* Ct <26.5. MRD reduction required a reduction in *NPM1* mutated transcripts of ≥1 log_10_ compared with pretreatment levels. MRD progression required an increase in *NPM1* mutated transcripts of ≥1 log_10._ Morphological relapse required the reappearance of >5% blasts in blood or bone marrow or extramedullary disease. Patients not meeting any of these criteria were designated to have stable disease. The overall response rate included patients who met the criteria for either MRD negativity or MRD reduction.

### Outcome measures

Overall survival (OS) was measured from day 1 of initiation of treatment to the date of death from any cause. Event-free survival (EFS) was measured from day 1 of treatment to the date of treatment failure, molecular or hematologic relapse, or death from any cause, whichever occurred first. Molecular relapse-free survival (RFS) was calculated only for patients achieving molecular response and defined as the time from the date of achievement of response until the date of molecular or hematologic relapse or death from any cause. In patients who ceased treatment, it was measured from the date of treatment cessation until the date of molecular or hematologic relapse or death from any cause. Patients not known to have relapsed or died at last follow-up were censored on the date they were last known to be alive.

### Statistical analysis

Quantitative variables were compared using Mann-Whitney *U* test or Kruskal-Wallis test and categorical variables using χ^2^ test. A 2-sided *P* value <.05 was considered statistically significant. The Kaplan-Meier method was used to assess OS, EFS, and RFS. A time-dependent regression analysis was performed to evaluate the effect of allogeneic hematopoietic stem cell transplantation (HSCT), represented using the Simon-Makuch method. These analyses were done using tmerge() function from R survival package (v. 3.5-5) and RcmdrPlugin.EZR R package (v. 1.61). Receiver operating characteristic curve analysis was used to determine the optimal cutoff value (Youden Index) of MRD that best correlated with response.

## Results

**Figure 1.**
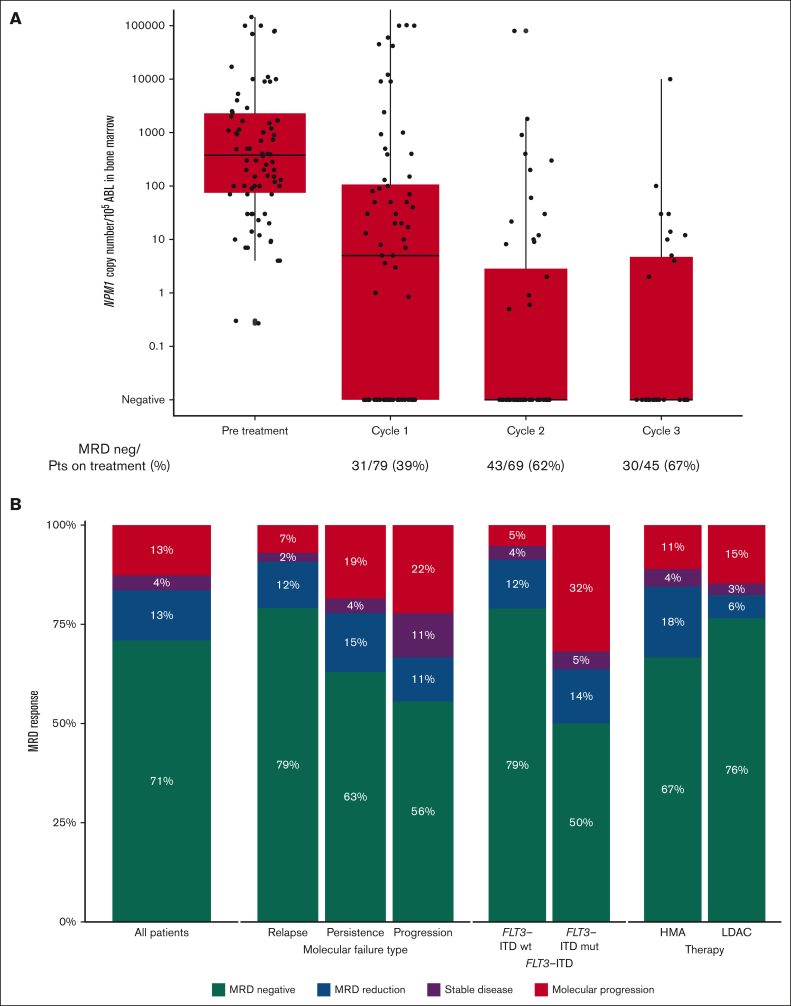
**MRD response.** (A) MRD levels before treatment and after the first 3 courses of venetoclax combinations, expressed as *NPM1* copies per 10^5^*ABL* in bone marrow. (B) Response rates in the whole cohort and depending on type of molecular failure, *FLT3*-ITD status at diagnosis and type of low-intensity chemotherapy given with venetoclax.

We identified 79 patients (median age, 62; range, 18-81 years) meeting the inclusion criteria. Thirty-one of 79 patients (39%) had a *FLT3* mutation at diagnosis, of whom 22 (28%) had *FLT3*-ITD. Seven of 79 patients (9%) had received a prior allograft (Table 1). The type of molecular failure was MRD relapse in 52 patients (66%, comprising 43 patients with molecular relapse and 9 with molecular progression) and molecular persistence in 27 (34%). Among the 27 patients treated for MRD persistence, 19 of 27 (70%) had only 1 risk factor for molecular progression (EOT *NPM1*^mut^ MRD <4.4-log reduction and were *FLT3*-ITD wild type), and 8 (30%) also had *FLT3*-ITD mutation. The median time from diagnosis of AML to molecular failure was 11 (range, 1-98) months, and the median level of MRD before treatment was 378 *NPM1* copies per 10^5^ *ABL* (range, 0.27-1 410 000; Table 1, Figure 1A).

**Table 1.** **Baseline characteristics of patients according to molecular failure**

	All cohort (N = 79)	Molecular relapse (n = 43)	Molecular persistence (n = 27)	Molecular progression (n = 9)	*P* value
Age, median (range), y	62 (18-81)	66 (18-81)	62 (31-77)	59 (30-70)	ns
Male, n (%)	42 (53)	24 (56)	12 (44)	6 (67)	ns
**AML type, n (%)**					
De novo	73 (92)	36 (84)	27 (100)	9 (100)	ns
Mutated FLT3-ITD	22 (28)	9 (21)	9 (33)	4 (67)	ns
Previous allogeneic HSCT, n (%)	7 (9)	5 (12)	0 (0)	2 (22)	<.01
Time from diagnosis to MRD relapse, median (range), mo	11 (1-98)	14 (5-98)	4 (1-18)	11 (6-16)	ns
Venetoclax dose (mg), median (range)	100 (70-600)	100 (70-400)	100 (100-400)	100 (100-600)	ns
MRD at relapse (NPM1 copies/10^5^ ABL), median (range)	378 (0.27-1 410 000)	495 (0.27-1 410 000)	150 (4-10 900)	9 000 (4-1 080 000)	<.01
**Venetoclax combination, n (%)**					
AZA	44 (56)	25 (58)	15 (56)	4 (44)	ns
LDAC	34 (43)	17 (40)	12 (44)	5 (56)	
DEC	1 (1)	1 (2)	0 (0)	0 (0)	
Antifungal prophylaxis∗, n (%)	53 (67)	30 (70)	16 (59)	7 (78)	ns
Number of cycles, median (range)	3 (1-25)	4 (1-23)	2 (1-25)	2 (1-14)	ns
Time between cycles, median (range), d	33 (19-69)	35 (19-62)	33 (27-69)	29 (26-32)	ns
**Response**					
MRD negativity, n (%)	56 (70.9)	34 (79.1)	17 (63)	5 (55.6)	ns
MRD reduction, n (%)	10 (12.7)	5 (11.6)	4 (14.8)	1 (11.1)	ns
No response, n (%)	13 (16.5)	4 (9.3)	6 (22.2)	3 (33.3)	ns
ORR (MRD negativity or reduction), n (%)	66 (83.5)	39 (90.7)	21 (77.8)	6 (66.7)	
Time to best MRD response, days, median (range)	56 (14-724)	54 (14-389)	77 (16-724)	47 (31-83)	<.01
Received HSCT, n (%)	44 (56)	28 (65)	12 (44)	4 (44)	ns
MRD negative before HSCT, n (%)	25 (32)	18 (42)	5 (15)	2 (22)	ns

AZA, azacitidine; DEC, decitabine; ns, not significant; ORR, overall response rate.

∗ Posaconazole, voriconazole, isavuconazole, and fluconazole were used as antifungal prophylaxis according to each center policy.

Patients were treated under institutional protocols using off-label venetoclax in combination with azacitidine (44/79 patients [56%]), LDAC (34/79 patients [43%]), or decitabine (1 patient). Azole antifungal prophylaxis was used in 48 patients (66%) with appropriate venetoclax dose reductions when indicated (Table 1). Patients received a median of 3 cycles (range, 1-25), with a median time between cycles of 32 days.

### MRD response

The median time from initiation of therapy to best MRD response was 56 days (range, 14-724). Three responding patients had an initial reduction in MRD but only achieved MRD negativity after >12 months of treatment.

Overall, MRD negativity was achieved in 56 of 79 patients (71%), and a molecular response (≥1-log reduction in MRD level) was observed in a further 10 of 79 (13%) for an overall molecular response rate of 84%. MRD negativity was achieved in 34 of 43 patients (79%) treated for molecular relapse, 17 of 27 (63%) of those treated for molecular persistence, and 5 of 9 (56%) of those treated for molecular progression. Molecular response was achieved in 39 (91%), 21 (78%), and 6 (67%) patients, respectively (Table 1). Three patients had been previously exposed to venetoclax combinations, 2 of them reached MRD negativity, and 1 progressed despite treatment.

Similar response rates were found irrespective of the combination regimen, with molecular responses observed in 84% of patients treated with azacitidine, 82% with LDAC, and 100% with decitabine (Figure 1B). Patients who had received a previous allogeneic HSCT had similar rates of response (5/7 [71%]) compared with those who had not (61/72 [85%]).

A pretreatment cutoff value of 365 copies of *NPM1*/10^5^ *ABL* at relapse was determined to be the most discriminative predictor of response (supplemental Figure 3). Patients with >365 copies of *NPM1*/10^5^ *ABL* were less likely to achieve a response (MRD negativity or reduction) with venetoclax combinations (odds ratio, 4.00; 95% IC, 1.08-15.8). Despite a lower response rate in patients with ≥365 copies of *NPM1*/10^5^ *ABL* before treatment, there were no differences in OS or EFS (supplemental Figure 4). Patients with MRD levels below and above the stablished cutoff point of 365 *NPM1*/10^5^ *ABL* copies proceeded to HSCT at similar rates (38.5% vs 50%, respectively; *P* = .31) (data not shown).

### Comutational landscape

Next-generation sequencing data at diagnosis were available for 73 of 79 patients. In this cohort, the most common co-occurring mutations were *DNMT3A* (34/79 [43%]), *FLT3*-ITD (22/79 [28%]), and *IDH2* (17/73 [23%]). Patients with *FLT3*-ITD mutations at diagnosis showed a lower response rate to venetoclax combinations (64% vs 91% in wild type; *P* = .005). We did not observe any differences in response rate or outcome according to *DNMT3A*, *IDH1/2*, or *RAS* pathway mutational status at diagnosis (Table 2).

**Table 2.** **Cox proportional hazard model univariable analysis for OS and EFS in all patients**

	OS, HR (95% CI)	EFS, HR (95% CI)
Age	0.99 (0.96-1.02)	0.98 (0.96-1.00)
AML (other vs de novo)	3.58 (1.20-10.7)	1.87 (0.8-4.39)
DNMT3A mutated vs wild type	0.79 (0.45-1.37)	0.95 (0.7-1.29)
FLT3-ITD mutated vs wild type	2.50 (1.06-5.86)	1.87 (1.06-3.28)
N/KRAS mutations vs wild type	1.23 (0.28-5.38)	0.59 (0.18-1.90)
IDH1/2 mutations vs wild type	1 (0.41-2.42)	1.33 (0.76-2.33)
Molecular progression vs molecular persistence	1.54 (0.38-1.75)	0.71 (0.27-1.89)
Molecular relapse vs molecular persistence	2.09 (0.79-5.58)	1.13 (0.63-2.01)
More than 365 NPM1 copies /10^5^ ABL at relapse (vs ≤365 copies)	1.51 (0.64-3.53)	1.01 (0.59-1.72)
Venetoclax combination (LDAC vs AZA)	0.87 (0.38-6.16)	0.86 (0.50-1.49)
Allogeneic HSCT after treatment vs no HSCT	1.28 (0.52-3.16)	0.81 (0.43-1.56)

AZA, azacitidine.

### Adverse events

Grade 4 neutropenia and thrombopenia were reported in 52 (66%) and 21 patients (27%), respectively. Eighteen patients required unplanned hospitalization due to febrile neutropenia, and 2 patients were admitted to critical care during treatment; 1 of them due to severe acute respiratory syndrome coronavirus 2 infection (supplemental Table 2). No deaths were reported during treatment.

### Outcomes

**Figure 2.**
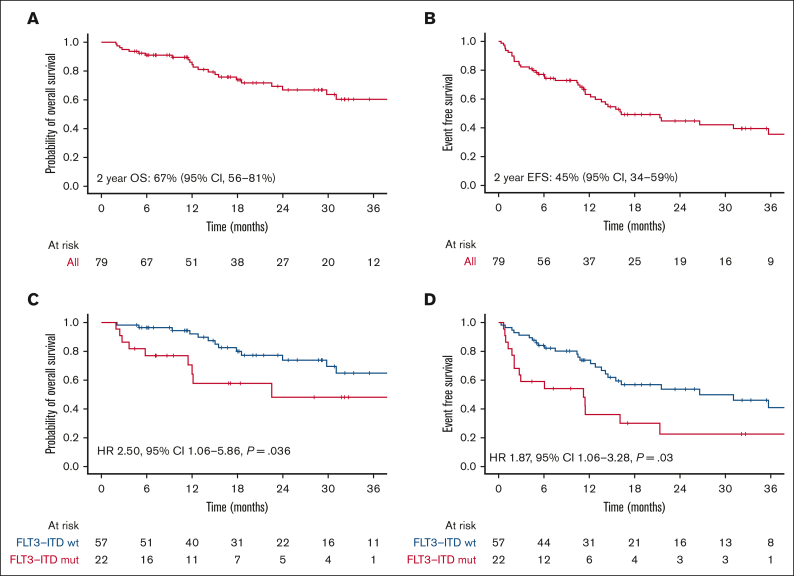
**Outcomes in the whole cohort and by *FLT3*-ITD status.** (A) OS and (B) EFS in patients treated with venetoclax combinations. (C) OS and (D) EFS depending on *FLT3*-ITD status at diagnosis.

With a median follow-up of 17 months (range, 2-64), 2-year OS was 67%, and 2-year EFS was 45%, with a median EFS of 16 months (Figure 2A-B). We found no differences in outcomes regardless of the treatment used or the type of MRD failure. The presence of *FLT3*-ITD mutation at diagnosis was associated with inferior OS (hazard ratio [HR], 2.50; 95% confidence interval [CI], 1.06-5.86; *P* = .036) and EFS (HR, 1.87; 95% CI, 1.06-3.28; *P* = .03) (Table 2, Figure 2C-D).

**Figure 3.**
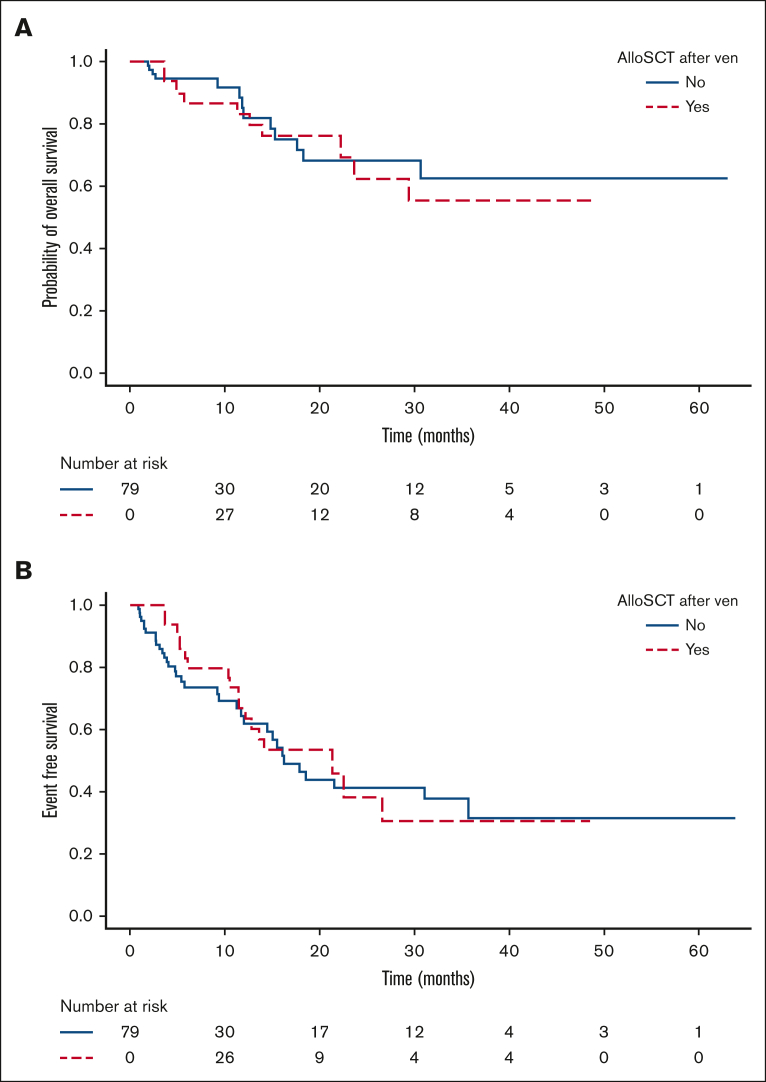
**Time-dependent regression analysis using the Mantel-Byar test to evaluate the impact of allogeneic HSCT on OS (A) and EFS (B).**

Forty-four of 79 patients (56%) underwent allograft at a median time from diagnosis of molecular failure of 5.2 months (range, 1-13.5), including 41 of 44 (93%) without further therapy, of whom 25 of 41 (57%) were MRD negative before transplant. In these 41 patients, allogeneic transplant did not have an impact on OS (HR, 1.28; 95% CI, 0.52-3.16; *P* = .6) or EFS (HR, 0.81; 95% CI, 0.43-1.56; *P* = .5) compared with those who did not undergo transplantation (Table 2, Figure 3A-B). Three patients underwent transplantation after subsequent treatment with FLAG-Ida-venetoclax (n = 1),^19^ FLAG-Ida (n = 1), and gilteritinib (n = 1) due to lack of response.

Among the 41 patients who proceeded to HSCT without any additional therapy, MRD negativity before HSCT did not have an impact on OS (median OS, not reached vs 21 months in MRD positive; *P* = .31) or EFS (median, 18 vs 12 months in MRD positive; *P* = .42) (supplemental Figure 5). Cumulative incidence of relapse at 12 months after transplant was 28% (supplemental Figure 6).

### Cessation of treatment

**Figure 4.**
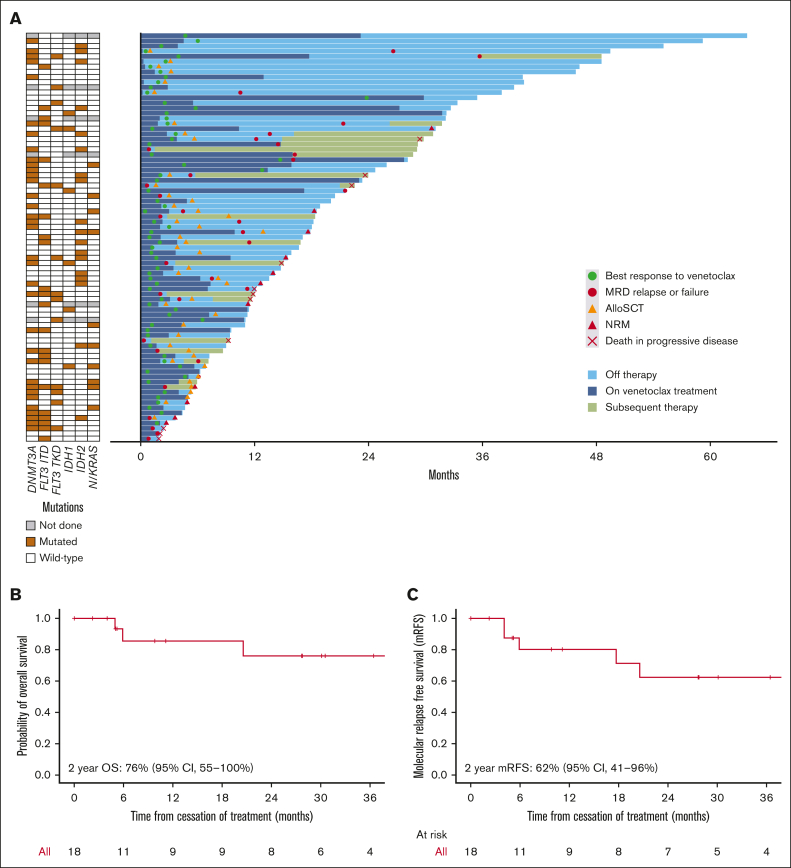
**Comutations, follow-up, and outcomes after cessation of treatment.** (A) Mutated genes at diagnosis and swimmer plot showing time of venetoclax treatment, response, and time off treatment. (B) OS and (C) molecular RFS in patients who ceased treatment after MRD negativity.

Nineteen patients who achieved a molecular response (18 of whom who achieved MRD negativity) and did not proceed to transplant electively ceased treatment after a median of 10 cycles (range, 2-30). Two-year OS was 76% in the 18 patients who were MRD negative at the time of treatment cessation, and 2-year molecular RFS was 62% (Figure 4B-C). Of note, only 3 (16%) of these patients had a *FLT3*-ITD mutation.

## Discussion

To our knowledge, this is the largest report to date evaluating the efficacy of low-intensity chemotherapy combined with venetoclax for *NPM1* molecular failure. The efficacy of SC has been demonstrated before in this subset of patients; for example, in the NCRI AML17 trial, 27 patients with molecular relapse received SC, and 16 (59%) achieved MRD negativity.^9^ In the CETLAM group cohort, 10 of 33 patients with molecular failure received SC (FLAG-IDA, HiDAC), and 80% achieved MRD negativity.^20^ In the VALDAC study, patients received LDAC and venetoclax after MRD or oligoblastic relapse (defined as <15% bone marrow blasts); of those with *NPM1*^mut^ AML, 11 of 20 patients (55%) with MRD relapse, and 6 of 8 with oligoblastic relapse achieved MRD negativity.^21^

Although venetoclax combinations have been reported to have particular efficacy in *NPM1*^mut^ AML,^22^ the response rate (complete remission + complete remission with incomplete count recovery) for frank hematologic relapse was only 46% in a retrospective study.^23^ Responses are similar with these combinations when patients relapse after HSCT, but in 1 report, 2 of 2 patients with *NPM1*^mut^ with molecular relapse had a sustainable remission.^24^

Here, we report molecular complete remission rates of 56% to 79% (depending on the type of failure), and this is consistent with previous smaller studies in the molecular failure setting reporting MRD negativity in 82% to 92% of patients.^13^^,^^14^ MRD negativity rates were similar to those reported with SC, despite the much higher toxicity and health care resource use associated with the latter.^9^^,^^20^ We found a rapid response to venetoclax, with best response achieved in more than half of the patients before the third cycle, consistent with previous literature.^13^^,^^25^^,^^26^ Three patients had an initial molecular response but only achieved MRD negativity after >12 months of treatment. Of note, this is consistent with a previous report in patients with newly diagnosed AML treated with firstline azacitidine-venetoclax, in which 21% of patients who achieved negative MRD by flow cytometry did so after >7 cycles.^27^

A previous publication found that patients with *NPM1*^mut^ AML who have positive MRD at EOT have a heterogeneous evolution, with a 1-year EFS <50% in patients with failure to clear MRD below 4.4 log_10_ from baseline and/or *FLT3*-ITD mutation.^17^ The benefit and optimal timing of treatment for these patients is not well determined, so, only those with ≥1 of these risk factors for progression were included in this cohort. Consistent with previous studies, we found worse OS and EFS in the presence of both risk factors, despite treatment with venetoclax combinations. Nonetheless, whether these patients benefit from therapy needs to be determined in prospective trials.

*FLT3*-ITD mutation, previously described as a marker of worse response to venetoclax,^22^^,^^28^^,^^29^ was also associated with a lower response rate in our cohort. AML harboring *K/NRAS* mutations have shown an intermediate response to venetoclax combinations,^23^^,^^28^ whereas patients with *IDH* mutations appear to have superior outcomes.^30^^,^^31^ We did not find differences in responses or outcomes according to *K/NRAS* or *IDH*1/2 mutational status in this cohort, although the limited patient numbers preclude any definite conclusions regarding these molecular subgroups.

Allogeneic transplant did not result in improved OS or EFS in this cohort. Although the decision to proceed with HSCT and when to do so was individual, and both the cohort size and length of follow-up are relatively limited, these data raise the question of the potential benefit of HSCT in patients with molecular failure treated with venetoclax-based combinations. However, addressing this question will require a randomized study. In contrast to previous reports^9^^,^^10^ for patients proceeding to HSCT, pretransplant MRD did not have an impact on outcome. This discrepancy may be related to the relatively small cohort and relatively short follow-up after HSCT. There were insufficient data available regarding conditioning intensity to evaluate the impact of this in patients with MRD positivity.^9^^,^^32^

Eighteen patients who ceased treatment after achieving MRD negativity had good outcomes, with molecular RFS at 4 years of 62%. A previous report showed that patients treated with frontline venetoclax combinations who achieved MRD negativity had *NPM1* or *IDH2* mutations, and those who discontinued treatment after 12 months had a median RFS of 59 months.^33^ Our results indicate that the option of treatment cessation has comparable outcomes after MRD relapse in *NPM1*^mut^ AML treated with venetoclax combinations.

In this cohort, the rate of adverse events including hematologic toxicity was low, and the toxicity profile appeared more favorable than with frontline therapy with venetoclax-based regimens, due to a lower incidence of febrile neutropenia (23% vs 42%).^26^^,^^34^

The main limitation of this study is its retrospective nature and patient recruitment, influenced by the availability of off-label venetoclax treatment, which may have induced a selection bias. Furthermore, being a multicenter cohort, the method used for MRD assessment, although standardized, may have introduced some differences.

Given the diverse treatment strategies used in this retrospective study, ranging from a finite number of venetoclax-based courses to consolidation with an allogeneic transplant, the optimal consolidation strategy in patients achieving a molecular complete remission is uncertain and should be addressed in future prospective studies. A phase 2, nonrandomized trial to assess the efficacy of azacitidine-venetoclax as a bridge to HSCT in *NPM1* molecular failure is currently active (www.clinicaltrials.gov identifier #NCT04867928).

Conflict-of-interest disclosure: A.M.-R. reports consultancy or advisory role in Bristol Myers Squibb (BMS), AbbVie, and Kite Gilead; travel grants from Kite Gilead, 10.13039/100004337Roche, Takeda, 10.13039/100005565Janssen, and 10.13039/100006483AbbVie; and speaker fees from AbbVie and Gilead. A.H.W. has served on advisory boards for Novartis, AstraZeneca, Astellas, Janssen, Jazz, Amgen, Roche, Pfizer, AbbVie, Servier, Gilead, BMS, and BeiGene; has consulted for AbbVie, Servier, Novartis, Shoreline, and Aculeus; receives research funding to the institution from 10.13039/100004336Novartis, 10.13039/100006483AbbVie, 10.13039/501100011725Servier, BMS, 10.13039/100018201Syndax, 10.13039/100013870Astex, 10.13039/100004325AstraZeneca, and 10.13039/100002429Amgen; serves on speaker’s bureaus for AbbVie, Novartis, BMS, Servier, and Astellas; is an employee of the Walter and Eliza Hall Institute (WEHI), and WEHI receives milestone and royalty payments related to the development of venetoclax; current and past employees of WEHI may be eligible for financial benefits related to these payments, and A.H.W. receives such a financial benefit. M. Jädersten has received institutional support from 10.13039/100006483AbbVie for arranging educational webinars and has served on advisory boards for AbbVie. S.K. has served on advisory boards for Astellas, Jazz, AbbVie, Servier, and Novartis; speaker’s bureau of Astellas, Jazz, and Novartis; and research funding from 10.13039/100004336Novartis. D.T.K. received consulting/advisory fees from AbbVie, Atheneum, and Astellas Pharma. V.M. has provided consultancy and received speaker honorarium from AbbVie, Jazz, Novartis, and Pfizer, and educational grants from Astellas and Takeda. N.O. has served on advisory boards for Takeda and Jazz; has consulted for AbbVie, Astellas, BMS, and Servier; and has received support for conference registration/accommodation/travel costs from AbbVie, Jazz, Pfizer, Servier, and Takeda. A.S.R. has provided consultancy to AbbVie and received travel grants from 10.13039/100011096Jazz Pharmaceuticals. The remaining authors declare no competing financial interests.
