# Supplementary material for: Venetoclax–based low intensity therapy in molecular failure of NPM1-mutated AML
Source: Blood Adv. 2023 Dec 2;8(2):343–52. doi: 10.1182/bloodadvances.2023011106 (PMC10788851; doi:10.1182/bloodadvances.2023011106)

Supplementary Appendix to:

Venetoclax-based low intensity therapy in molecular failure of *NPM1*-mutated AML.

Carlos Jimenez-Chillon[1,2], Jad Othman[2,-4], David Taussig[5], Carlos Jimenez-Vicente[6], Alexandra Martinez-Roca[6,7], Ing Soo Tiong[8-10], Manish Jain[11], James Aries[12], Seda Cakmak[12], Steven Knapper[13], Daniel Tuyet Kristensen[14], Vidhya Murthy[15], Joy Zacharoula Galani[16], Charlotte Kallmeyer[17], Loretta Ngu[18], David Veale[18], Simon Bolam[19], Nina Orfali[20], Anne Parker[21], Cara Manson[21], Jane Parker[22], Thomas Erblich[23], Deborah Richardson[24], Katya Mokretar[25], Nicola Potter[2], Ulrik Malthe Overgaard[26,27], Anne Stidsholt Roug[14,28] Andrew H. Wei[8], Jordi Esteve[7], Martin Jädersten[29,30], Nigel Russell[3], Richard Dillon[2,3].

1) Servicio de Hematología y Hemoterapia, Hospital Universitario Ramón y Cajal, Madrid, Spain. 2) Department of Medical & Molecular Genetics, King's College, London, United Kingdom. 3) Guy's and St Thomas Hospital, London, United Kingdom. 4) Faculty of Medicine and Health, University of Sydney, Australia. 5) Department of Haematology, Royal Marsden Hospital, Sutton, United Kingdom. 6) Institut d'Investigacions Biomèdiques August Pi i Sunyer (IDIBAPS), Barcelona, Spain. 7) Hematology Department. Hospital Clínic Barcelona, Spain. 8) Peter Mac Callum Cancer Centre, Royal Melbourne Hospital and Walter and Eliza Hall Institute of Medical Research, Melbourne, Vic., Australia. 9) Alfred Hospital and Monash University, Melbourne, VIC, Australia. 10) Austin Health and Olivia Newton John Cancer Research Institute, Melbourne, VIC, Australia. 11) Department of Haematology, Leeds Teaching Hospitals Trust, Leeds, United Kingdom. 12) Department of Haemato-Oncology, St Bartholomew's Hospital, London, United Kingdom. 13) Department of Haematology, School of Medicine, Cardiff University, Cardiff, United Kingdom. 14) Department of Haematology, Clinical Cancer Research Center, Aalborg University Hospital, Aalborg. 15) Department of Haematology, University Hospitals of Birmingham, United Kingdom. 16) Department of Haematology, Dartford & Gravesham NHS Trust, Dartford, United Kingdom. 17) Department of Haematology, Lincoln County Hospital, Lincoln, United Kingdom. 18) Department of Haematology, Royal Devon University Healthcare NHS Foundation Trust, Exeter, Devon, United Kingdom. 19) Department of Haematology, Taunton and Somerset NHS Foundation Trust, Taunton, United Kingdom. 20) Department of Haematology, St. James's Hospital, Dublin, Ireland. 21) Department of Haematology, Queen Elizabeth University Hospital, Glasgow, United Kingdom. 22) Department of Haematology, Northampton General Hospital, Northampton, United Kingdom. 23) Department of Haematology, The London Clinic, London, United Kingdom. 24) Department of Haematology, University Hospital Southampton, Southampton, United Kingdom. 25) Synnovis, London, United Kingdom. 26) Department of Haematology, Rigshospitalet, Copenhagen, Denmark. 27) Department of Haematology, National Hospital, Copenhagen, Denmark. 28) Department of Hematology, Aarhus University Hospital, Aarhus, Denmark. 29) Center for Haematology and Regenerative Medicine, Department of Medicine, Karolinska Institutet, Stockholm, Sweden. 30) Department of Haematology, Karolinska University Hospital, Stockholm, Sweden

Address for correspondence:
Dr Richard Dillon, Department of Medical and Molecular Genetics, King's College, London
Floor 7, Tower Wing, Guy's Hospital, London SE1 9RT, United Kingdom
Email: richard.dillon@kcl.ac.uk

Supplementary Tables:

Supplementary Table 1. Induction chemotherapy previous to molecular failure treated with venetoclax and low intensity chemotherapy.

**Previous chemotherapy**	**n**	**(%)**
DA/3+7 with GO	28	35.5
DA/3+7	27	34.2
3+5	11	13.9
2+5	6	7.6
CPX-351	4	5.1
IDAC	2	2.5
FLA-Ida	1	1.2
Previous chemotherapy combined with midostaurin	23	29.1

Supplementary Table 2. Incidence of adverse events during venetoclax treatment.

	**Molecular relapse (n=43)**	**Molecular persistence (n=27)**	**Molecular progression (n=9)**	**All cohort (n=79)**
Grade 4 neutropenia, n (%)	24 (57)	22 (81)	6 (67)	52 (66)
Grade 4 thrombopenia, n (%)	10 (24)	7 (26)	4 (44)	21 (27)
Febrile neutropenia, n (%)	11 (26)	5 (19)	2 (22)	18 (23)
Unplanned hospitalisation, n (%)	12 (28)	5 (19)	1 (11)	18 (23)
ICU admission, n (%)	2 (4.7)	0 (0)	0 (0)	2 (2.5)

Supplementary Figures:

Supplementary Figure S1. CONSORT diagram of patients included in the study.

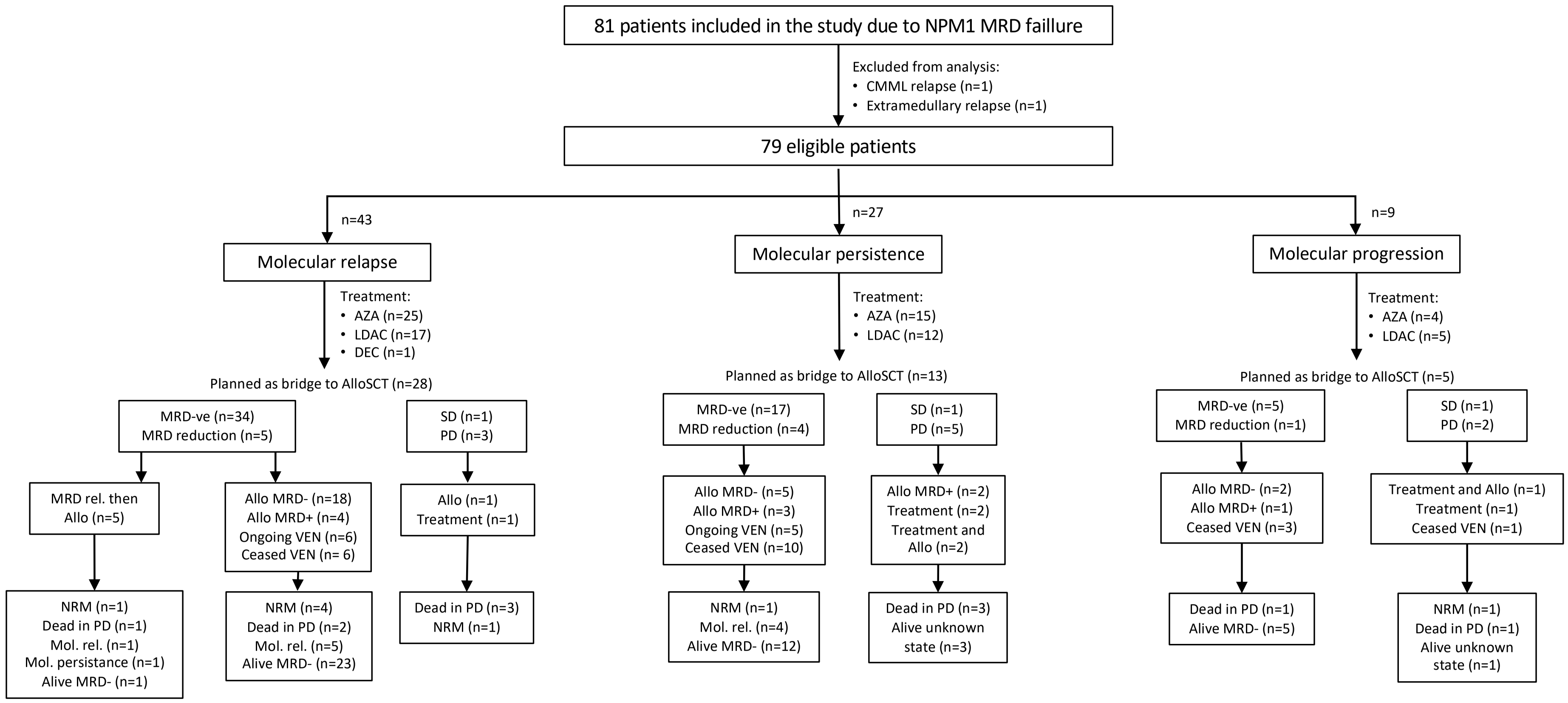

Supplementary Figure S2. Bone marrow *NPM1* copy number after each cycle of venetoclax treatment depending on reason of treatment.

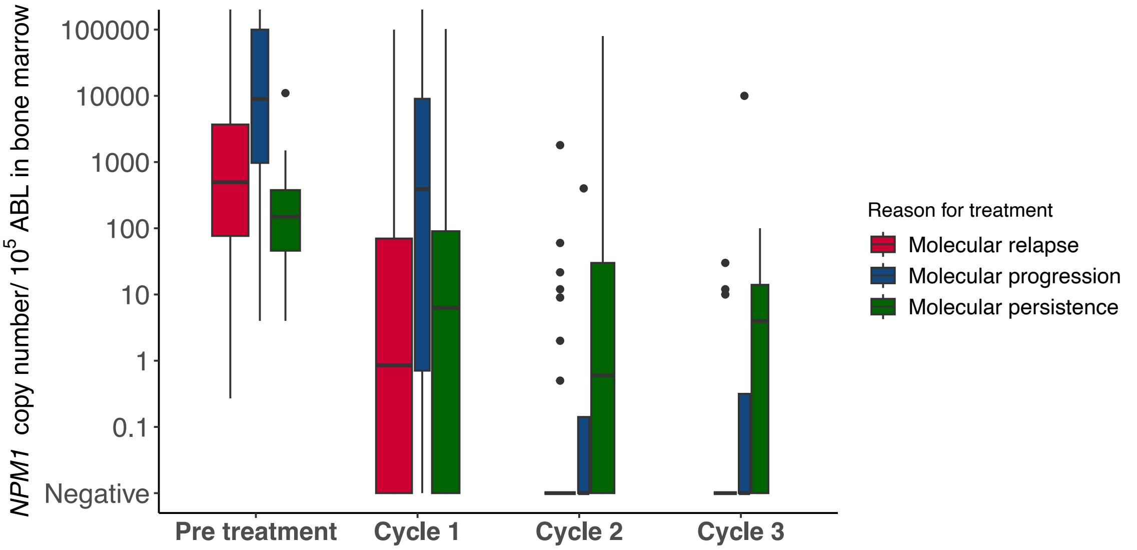

Supplementary Figure S3. (A) ROC curve to determine the point of MRD level at relapse with the higher sensitivity and specificity as a predictor of response to venetoclax combinations in our cohort. (B) Response rates according to pre-treatment MRD levels. OS (C) and EFS (D) depending on pre-treatment MRD levels (cut-off point of 365 *NPM1* copies / 10^5 *ABL*).

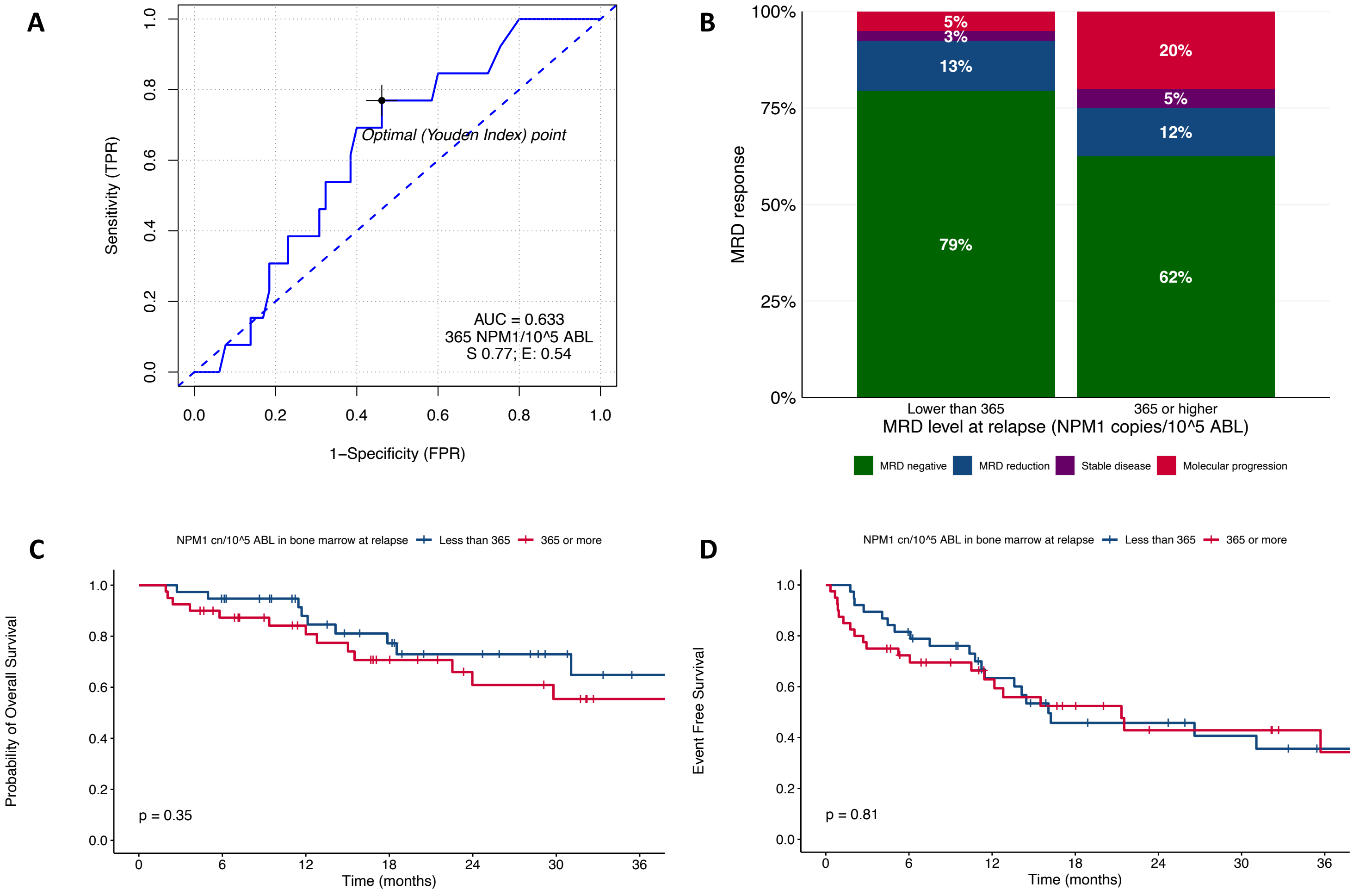

Supplementary Figure S4. Odds Ratio (OR) for response.

**Subgroup**	*no. /total (%)*	**OR for response (IC 95%)**
Previous allogeneic HSCT	7/79 (8.9)	2.22 (0.38-12.9)
FLT3 -TKD^{mut}	13/79 (16.5)	2.82 (0.71-11.1)
FLT3 -ITD^{mut}	22/79 (27.8)	5.9 (1.68-21.1)
IDH1/2 ^{mut}	24/73 (32.8)	0.56 (0.14-2.25)
N/KRAS ^{mut}	10/73 (13.7)	1.18 (0.22-6.34)
MRD at relapse >365 *NPM1* / 10^5AE	40/79 (50.6)	4 (1.08-15.8)

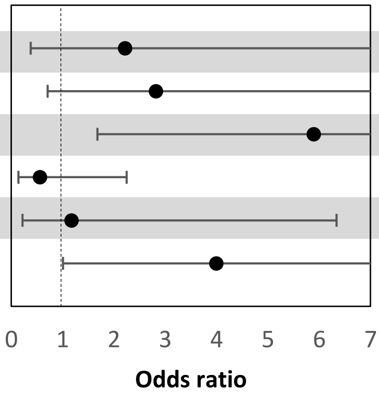

Supplementary Figure S5. OS (A) and EFS (B) in patients proceeding to allogeneic SCT after venetoclax-combinations without any further therapy, depending on pre-transplant MRD.

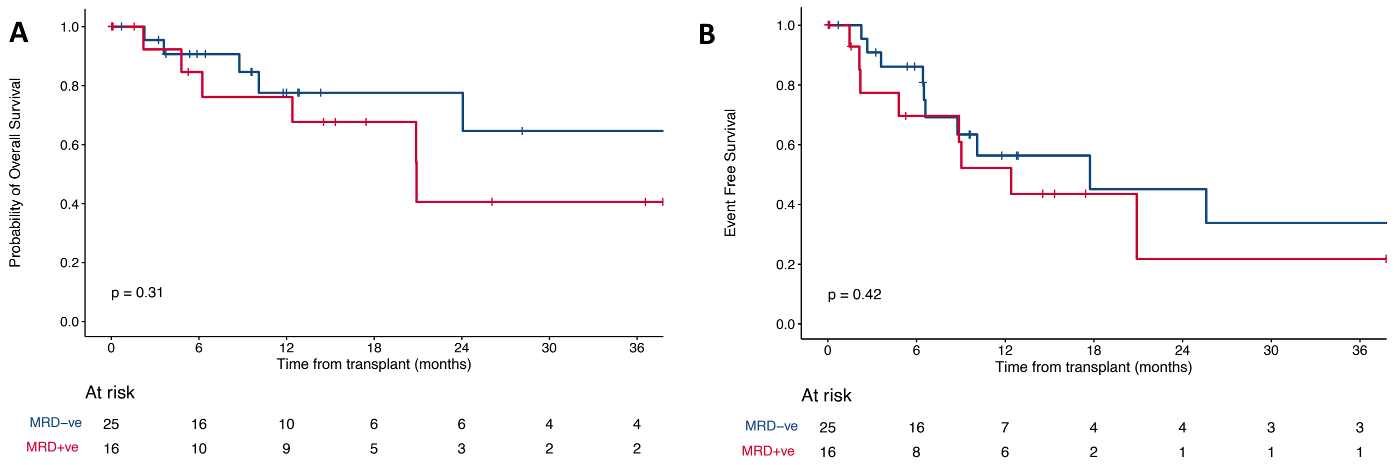

Supplementary Figure S6. Cumulative incidence of relapse (CIR) and death (CID) from time of transplant in patients proceeding to allogeneic HSCT in molecular response.

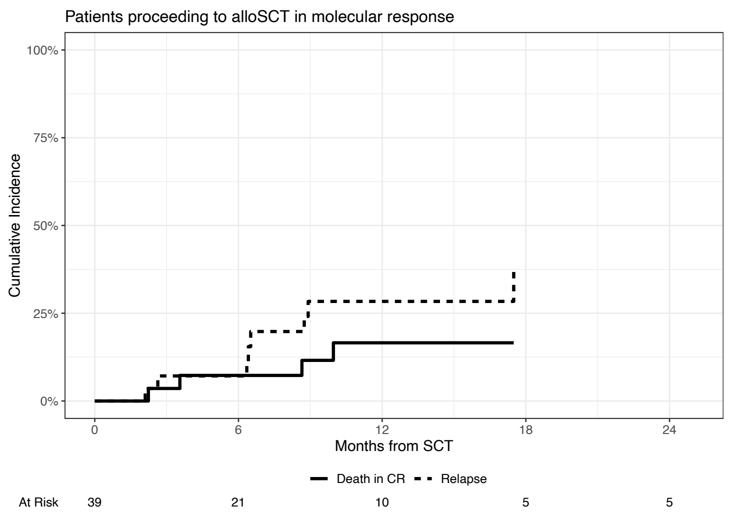

Supplement: Supplemental Tables and Figures [file BLOODA_ADV-2023-011106-mmc1.pdf]
